# Tumorigenic and Metastatic Role of CD44^−/low^/CD24^−/low^ Cells in Luminal Breast Cancer

**DOI:** 10.3390/cancers12051239

**Published:** 2020-05-14

**Authors:** Rajeev Vikram, Wen Cheng Chou, Shih-Chieh Hung, Chen-Yang Shen

**Affiliations:** 1 Taiwan International Graduate Program in Molecular Medicine, National Yang–Ming University and Academia Sinica, Taipei 115, Taiwan; rajeev.vkrm@ibms.sinica.edu.tw; 2 Institute of Biomedical Sciences, Taipei 115, Taiwan; wencheng@ibms.sinica.edu.tw; 3 Drug Development Center, Institute of New Drug Development, China Medical University, Taichung 404, Taiwan; hung3340@gmail.com; 4 Graduate Institute of Environmental Science, China Medical University, Taichung 404, Taiwan

**Keywords:** breast cancer, ALDH1A1, CD44, CD24, metastasis, ALDH

## Abstract

Cells with high CD44 but low CD24 expression (CD44^high^/CD24^−/low^) and high aldehyde dehydrogenase activity (ALDH^br^) are widely considered to be drivers of metastasis, therapy resistance and tumor recurrence in breast cancer. However, the role of the CD44^high^/CD24^−/low^ and ALDH^br^ phenotypes in identifying tumorigenic cells in breast cancer remains controversial due to the discrepancy in their distribution and tumorigenic potential in intrinsic breast cancer subtypes. In this study, we analyzed the cells expressing these markers in six different breast cancer cell lines representing major breast cancer subtypes (T47D, MCF-7, BT-474, AU-565, Hs578T and MDA-MB-231). CD44^high^/CD24^−/low^, ALDH^br^ and CD44^−/low^/CD24^−/low^ cell populations were isolated by flow cytometry and analyzed for hallmark stem cell characteristics of differentiation, migration, invasiveness and metastasis using in vitro and in vivo techniques. Our results demonstrate that the CD44^−/low^/CD24^−/low^ cell population, which is enriched in luminal cell lines (T47D, MCF-7 and BT-474), possesses metastatic and tumorigenic properties. We also show that, contrary to previous claims, the expression of the ALDH1 isoform ALDH1A1 does not affect the tumorigenic potential of cell lines with high ALDH activity (BT-474 and AU-565). Further transcriptomic and clinical studies are needed to determine the potential of these markers as early diagnostic tools and treatment targets.

## 1. Introduction

Breast cancer is the most prevalent type of malignancy in women worldwide, leading to thousands of deaths every year. Based on the presence or absence of commonly evaluated hormone receptors—estrogen (ER), progesterone (PR) and human epidermal growth factor 2 (HER2)—breast cancer is divided into four main subtypes: luminal A (ER+/PR+/HER2−), luminal B (ER+/PR+/HER2+), HER2 overexpressing or HER2-OE (ER−/PR+/HER2+) and triple-negative/basal-like type (ER−/ER−/HER2−) [1].

According to the cancer stem cell (CSC) hypothesis, irrespective of the molecular subtypes, breast cancer cells can evade traditional therapy and are able to enter a metastatic state because of a small population of tumorigenic cells that possess a stem/progenitor cell-like capacity of self-renewal and differentiation. These tumorigenic cells are also referred to as breast cancer stem cells (BCSCs). This tumorigenic subpopulation is considered to be responsible for intratumoral heterogeneity, drug resistance and tumor recurrence [2]. CSC identification has been largely driven by a combination of different cellular markers that are considered to be CSC specific. Indeed, this hypothesis is supported by data obtained for solid tumors from a number of carcinomas and sarcomas, including brain [3], liver [4], oral [5], colon [6], osteosarcoma [7], rhabdomyosarcoma [8], and Ewing’s sarcoma [9].

For breast cancer, Al−Hajji et al. (2003) [10] showed that cells which overexpress the transmembrane glycoprotein CD44 and underexpress the cell membrane sialoglycoprotein CD24 (CD44^high^/CD24^−/low^), possess tumorigenic properties. Since that discovery, the CD44^high^/CD24^−/low^ subpopulation has been found in other carcinomas such as squamous cell carcinoma of the oral cavity and head/neck region [11].

Aldehyde dehydrogenase (ALDH) is another biomarker that has been widely used for characterizing cell stemness [12]. The ALDH superfamily is associated with alcohol metabolism, retinoic acid (RA) metabolism and protection from reactive oxygen species [13]. ALDH-dependent RA signaling is implicated in morphogenesis, cell development and stem cell regulation [12]. Cells with increased ALDH activity (ALDH^br^) have tumorigenic behavior in breast cancer [14]. A few studies have also suggested that a very small subpopulation of cells that overexpress both CD44 and ALDH (CD44^high^/CD24^−/low^/ALDH^br^) may be potent initiators of breast tumorigenesis [15,16].

Recent studies, however, have shown that the phenotypes identified by these markers do not universally correlate with tumorigenic cells in different molecular subtypes. Studies indicate that the ALDH^br^ subpopulation is pronounced in the HER2-OE, whereas the CD44^high^/CD24^−/low^ subpopulation is more apparent in the triple-negative/basal-like subtype [17]. The luminal subtypes, which constitute the majority of breast cancer cases, are predominantly CD44^low^/CD24^–/low^/ALDH^–/low^. It remains unclear whether CD44^high^ or ALDH^br^ cells play a tumorigenic role in these breast cancer subtypes [17,18]. In contrast, CD44^–/low^/CD24^high^ cells have been observed in the majority of invasive breast carcinomas [19]. Moreover, in breast cancers with high ALDH activity, the expression of the ALDH1 isoform ALDH1A1 did not correlate with high metastasis or poor survival [20]. Some studies have also suggested that tumor cell metastasis may not depend exclusively on tumor cells that express these stemness-related markers [21]. More importantly, from a clinical perspective, ALDH1A1 overexpression does not significantly correlate with a poor clinical outcome in different cancers [21,22,23]. Overall, these studies present a contrasting picture of the correlation between the expression level of these markers and tumorigenicity in breast cancer cells.

Considering the conflicting nature of data regarding BCSC phenotypes, it is important to systematically investigate the expression of CD44, CD24 and ALDH1A1 in different breast cancer subtypes to understand whether the expression of these biomarkers indeed correlates with tumorigenic potential. Specifically, in luminal breast cancers, which constitute 70% of all breast cancers, it is critical to assess the tumorigenicity and BCSC-like properties of CD44^low^/CD24^–/low^/ALDH^–^ cells, because this subpopulation reflects the major luminal breast cancer phenotype.

In this study, we performed a systematic comparison of six breast cancer cell lines representing different molecular subtypes, including luminal A (T47D and MCF-7), luminal B (BT-474), HER2-OE (AU-565) and triple-negative/basal-like subtype (MDA-MB-231 and Hs578T) for CD44, CD24 expression and ALDH activity. We first categorized the cells into different subpopulations according to biomarker expression and then carried out a systematic and comparative analysis of these subpopulations for hallmark stem cell characteristics of differentiation, migration, invasiveness, and metastatic potential using established in vitro and in vivo techniques. We also analyzed the expression of genes that are considered to be hallmarks of metastasis, pluripotency and proliferation. Finally, we assessed the expression of ALDH1 isotypes ALDH1A1 and ALDH1A3 in these phenotypes to ascertain the role of ALDH1 in BCSC function.

## 2. Results

### 2.1. Luminal Subtypes Have a Predominantly CD44^−/low^/CD24^−/low^ Phenotype

**Figure 1:**
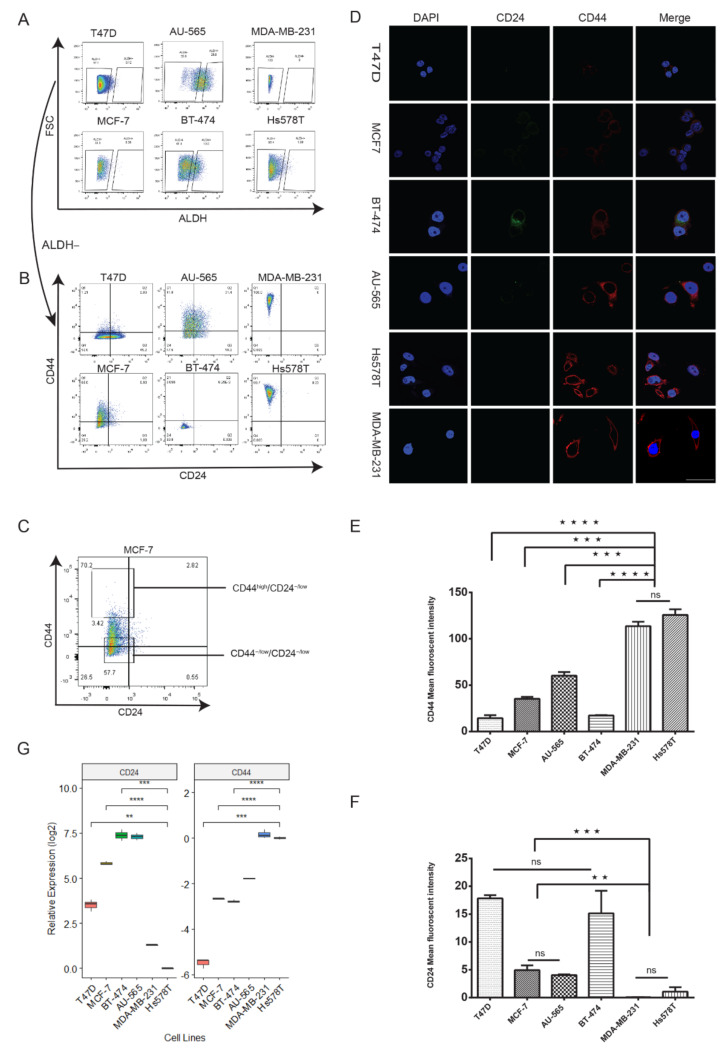
CD44, CD24 and ALDH expression in breast cancer cell lines is subtype dependent (**A**–**B**) Flow cytometry analysis of ALDH, CD44 and CD24 expression in different molecular subtypes of breast cancer. Cells were treated with ALDEFLUOR reagent and double stained with anti-CD44-APC (allophycocyanin) and anti-CD24-PerCP-eFluor 710. The accuracy of the double immunostaining was confirmed by comparison with single immunostaining. FSC—forward scatter. ALDH^br^ cells were the highest in HER2-OE cell lines, CD44^−/low^/CD24^−/low^ in luminal and CD44^high^/CD24^−/low^ in triple-negative cell lines (Supplementary Figure S1). (**C**) Representative gating for the selection of CD44^high^/CD24^−/low^ and CD44^−/low^/CD24^−/low^ cells. (**D**) Representative immunofluorescence images showing the expression of CD24 (green), CD44 (red) and DAPI (blue) in breast cancer cell lines. Cells were stained with primary anti-CD24 and anti-CD44 antibodies. Scale bar = 100 μm. (**E**–**F**) Comparison of average fluorescence intensities of CD44 and CD24. (**G**) The relative mRNA expression of CD24 and CD44 by qRT-PCR shows that luminal cell lines have a low expression of both CD44 and CD24. All mRNA expression compared to Hs578T. Data represent the mean ± SD of three independent experiments; ** *p* < 0.01, *** *p* < 0.001, and **** *p* < 0.0001.

To understand the relation of CD44, CD24 and ALDH1 expression with tumorigenic cells in breast cancer, we chose six cell lines to represent the major molecular subtypes of breast cancer: MCF-7 and T47D (luminal A), BT-474 (luminal B, HER2-OE), AU-565 (HER2-OE), Hs578T and MDA-MB-231 (triple-negative/basal like). Recent research has suggested that a high CD44/CD24 ratio and high ALDH expression correlate positively with proliferation and tumorigenesis [14,24,25,26]. Using flow cytometry, we investigated the expression of CD44, CD24 and ALDH activity in these cell lines. We analyzed ALDH activity by treating the cell lines with ALDEFLUOR and calculating the percentage of ALDEFLUOR-stained cells (ALDH^br^). ALDH^br^ cells were the highest in HER2-OE cell lines BT-474 (20%) and AU-565 (26%); the prevalence was <1% in all other lines (Figure 1A). However, we did not find ALDH^br^ cells in MDA-MB-231. Consistent with previous research [21,27], our results revealed that triple-negative cell lines had a primarily CD44^high^/CD24^−/low^/ALDH^−^ profile (CD44^high^/CD24^−/low^), whereas luminal A cell lines MCF-7 and T47D had a CD44^−/low^/CD24^−/low^/ALDH^−^ profile (CD44^−/low^/CD24^−/low^). Luminal B and HER2-OE cell lines BT-474 and AU-565 had a mixed profile of CD44^−/low^/CD24^−/low^ and CD44^−/low^/CD24^−/low^/ALDH^br^ (ALDH^br^) cells, with CD44^−/low^/CD24^−/low^ constituting the majority of cells (Figure 1B, Supplementary Figure S1A). Fluorescence and real-time quantitative reverse transcription PCR (qRT-PCR) analysis of CD44 and CD24 in the cell lines showed a similar expression profile (Figure 1D–G), with triple-negative cell lines significantly overexpressing CD44. Interestingly, MCF-7 showed a lower expression of CD44 compared to AU-565 (Figure 1E, G) which is considered to have comparatively lower malignancy [28]. Taken together, these results show that luminal A breast cancer cell lines (T47D and MCF-7) were predominantly CD44^−/low^/CD24^−/low^ and ALDH^−^. Cell lines overexpressing HER2 (BT-474 and AU-565) had a higher proportion of ALDH^br^ cells and triple-negative cell lines (Hs578T and MDA-MB-231) were almost exclusively CD44^high^/CD24^−/low^.

### 2.2. Lumina CD44^−/low/^CD24^−/low^ Cells Exhibit Properties of Self-Renewal and Differentiation

**Figure 2:**
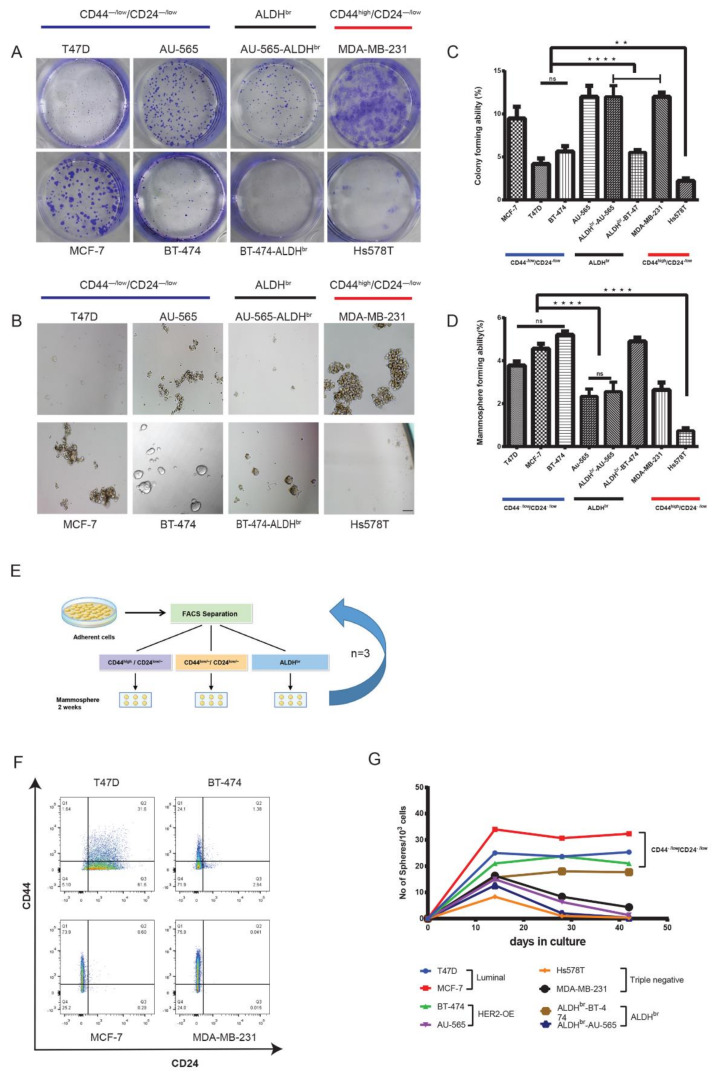
Luminal CD44^−/low^/CD24^−/low^ cells propagate as colonies and form long-term viable spheres. (**A**,**C**) Representative images and comparison of clonogenic assay and mammosphere assay (**B**,**D**) of the flow sorted CD44^−/low^/CD24^−/low^, CD44^high^/CD24^−/low^ and ALDH^br^ subpopulations from breast cancer cell lines. Spheres were counted after 7 days in culture (Supplementary Figure S2). (**E**) Schematic representation of long-term mammosphere assay. FACS-separated populations were seeded at a density of 2 × 10^4^ cells/mL in ultra-low-attachment plates and maintained as spheres. The whole procedure was repeated every 14 days, until 42 days (three generations). (**F**) FACS analysis of CD44^−/low^/CD24^−/low^, ALDH^br^ and CD44^high^/CD24^−/low^ mammospheres after three generations (42 days) shows a decrease in CD44 expression in mammospheres of CD44^high^/CD24^−/low^ origin from the MDA-MB-231 cell line. AU-565 and Hs578T cell lines could not form viable mammospheres after 7–12 days in culture. (**G**) Graph showing the number of viable mammospheres before each FACS separation. Spheres were counted under a microscope at 10× magnification. Data represent the mean ± SD of three independent experiments; ** *p* < 0.01 and **** *p* < 0. 0001. Scale bar = 200 μm.

Because the luminal and HER2-OE cell lines had a predominantly CD44^−/low^/CD24^−/low^ phenotype, we compared them with CD44^high^/CD24^−/low^ and ALDH^br^ cells with respect to tumorigenicity and stem cell-like characteristics in vitro and in vivo. A number of studies have reported that cells with the CD44^high^/CD24^−/low^ and ALDH^br^ phenotypes have greater tumorigenic potential than the CD44^−/low^/CD24^−/low^ phenotype [24,29]. To verify this assertion, we carried out sphere and colony formation assays. These assays have been widely applied to assess key stem cell characteristics of cell survival and the ability of a cell to undergo “unlimited” cell division in vitro [30,31,32]. We seeded the CD44^−/low^/CD24^−/low^, CD44^high^/CD24^−/low^ and ALDH^br^ subpopulations separated by flow cytometry at densities of 100, 500 and 1000 cells/well (in triplicate for each group) as monolayers and non-attached multicellular spheroids (mammospheres). CD44^high^/CD24^−/low^ cells from the MDA-MB-231 cell line had the highest colony forming ability (CFA), whereas CD44^high^/CD24^−/low^ cells from Hs578T had the lowest CFA. CD44^−/low^/CD24^−/low^ cells from luminal A and B cell lines showed significantly higher CFA than CD44^high^/CD24^−/low^ cells from Hs578T (Figure 2A,C). Mammosphere assay too showed a similar trend, with CD44^high^/CD24^−/low^ cells from Hs578T showing the lowest mammosphere-forming ability. ALDH^br^ cells from HER2-OE cell lines had a similar colony and mammosphere-forming ability to that of CD44^−/low^/CD24^−/low^ cells from their respective cell lines. However, both the CD44^−/low^/CD24^−/low^ and ALDH^br^ subpopulations from the HER2-OE cell line AU-565 had high colony forming but low mammosphere-forming ability (Figure 2B,D). Interestingly, only luminal cell lines (T47D, MCF-7 and BT-474) formed tightly bound or fused spheres (Supplementary Figure S2A).

Cells with tumorigenic or stem cell-like properties can survive and differentiate through multiple generations in a non-adherent environment [33]. Therefore, we compared the ability of CD44^−/low^/CD24^−/low^, ALDH^br^ and CD44^high^/CD24^−/low^ cells to retain their diversity and viability upon serial passage as mammospheres. While the viability of CD44^high^/CD24^−/low^ cells from MDA-MB-231 dropped substantially, CD44^−/low^/CD24^−/low^ cells from luminal cell lines (T47D, MCF-7 and BT-474) maintained viable mammospheres through three passages over 42 days (Figure 2G). After 7–10 days, the Hs578T and AU-565 cell lines could not be propagated further owing to low viability. The proportion of cells with the CD44^−/low^/CD24^−/low^ phenotype remained consistent in luminal cell lines (Figure 2F). As described previously, mammosphere assay enriches for tumorigenic cells [19], we therefore expected an increase in the proportion of the CD44^high^/CD24^−/low^ and/or ALDH^br^ phenotypes. Instead, the proportion of CD44^high^/CD24^–/low^ cells and the number of mammospheres in MDA-MB-231 decreased after the second passage (Figure 2F,G). A similar decrease in the proportion of CD44^high^/CD24^−/low^ cells and mammosphere-forming ability in MDA-MB-231 was observed by Wang et al. (2014) [34], indicating a decrease in the proliferative ability of MDA-MB-231-derived cells in long-term sphere culture. Taken together, these results show that ALDH^br^ cells from the HER2-OE cell line Au-565 and CD44^high^/CD24^–/low^ cells from triple-negative cell lines exhibit a decrease in survival and proliferative ability in sphere culture. Cells with CD44^−/low^/CD24^−/low^ phenotype from luminal cell lines exhibit consistent survival and retains the ability to differentiate and maintain subpopulation diversity, which are hallmark stem cell characteristics of differentiation and long-term survival.

### 2.3. Luminal CD44^−/low^/CD24^−/low^ Cells Express Key Pluripotency Regulators

**Figure 3:**
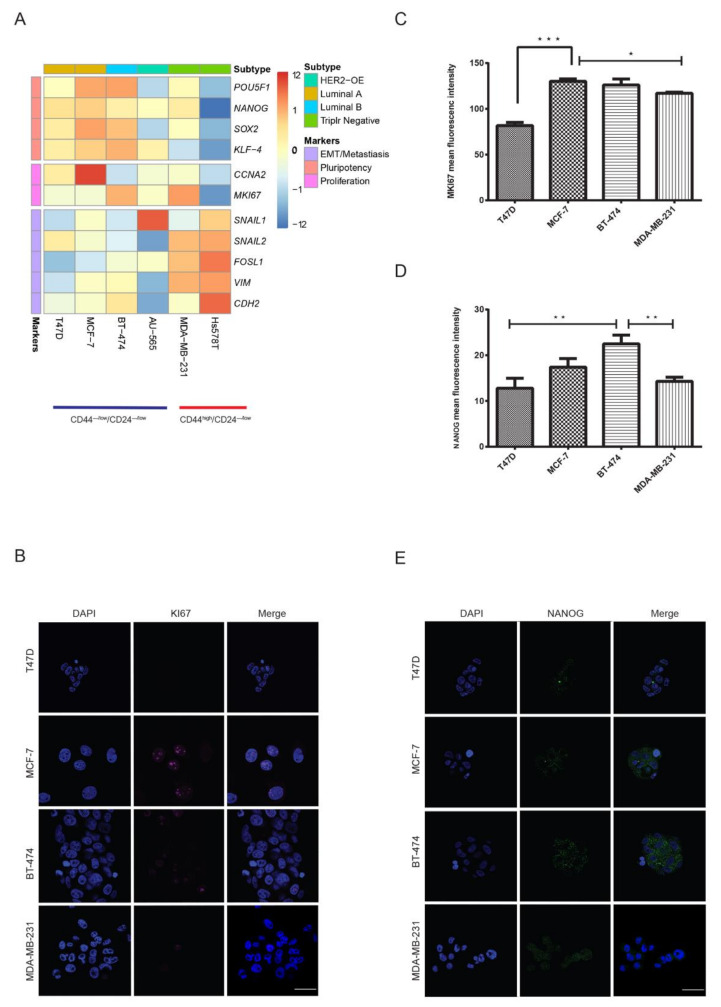
Luminal CD44^−/low^/CD24^−/low^ cells show a similar expression profile of pluripotency markers. Comparison of mRNA expression of markers for pluripotency, EMT/metastasis and proliferation for CD44^high^/CD24^−/low^ and CD44^−/low^/CD24^−/low^ cells, performed using qRT-PCR. (**A**) Heat map of genes with opposite expression patterns between CD44^high^/CD24^−/low^ and CD44^−/low^/CD24^−/low^ for pluripotency and EMT. Each row represents an RNA transcript; each column represents a cell line. CD44^−/low^/CD24^−/low^ cells from luminal cell lines overexpressed pluripotency markers (Supplementary Figure S3). Representative image and analysis of immunofluorescence for MKI67 (**B**,**C**) and NANOG (**D**,**E**) expression in mammospheres from CD44^−/low^/CD24^−/low^ and CD44^high^/CD24^−/low^ cells. Flow cytometry sorted cell populations were maintained as spheres for 14 days and stained with DAPI (blue), NANOG (green) and MKI67 (magenta). Spheres were observed under fluorescence microscope. Data represent the mean ± SD (n = 3) of three independent experiments; * *p* < 0.05, ** *p* < 0.01 and *** *p* < 0.001. Scale bar = 100 μm.

The lack of correlation between these markers and proliferative and survival capacity in the cell populations indicated differences in the expression of genes responsible for these characteristics. Therefore, we next compared the expression of key markers genes for pluripotency (*POU5F1, NANOG, SOX2, KLF-4)*, proliferation (*CCNA2, MKI67*) and epithelial-mesenchymal transition(EMT)/metastasis (*SNAIL1, SNAIL2, FOSL1,* vimentin *(VIM), CDH2*) between the CD44^−/low^/CD24^−/low^ (T47D, MCF-7 and BT-474, AU-565) and CD44^high^/CD24^−/low^ subpopulations (Hs578T and MDA-MB-231) using qRT-PCR. There was a clear distinction between the mRNA expression profiles of these genes between CD44^high^/CD24^−/low^ and CD44^−/low^/CD24^−/low^ cells. Broadly, CD44^−/low^/CD24^−/low^ cells from luminal cell lines (T47D, MCF-7 and BT-474) overexpressed pluripotency markers, whereas CD44^high^/CD24^−/low^ cells from triple-negative cell lines (Hs578T and MDA-MB-231) showed the overexpression of EMT/metastasis markers (Figure 3A). Specifically, the expression of NANOG and SOX2, which are demonstrated to drive tumorigenesis and metastasis in breast cancer [35,36], was 5-fold higher in CD44^−/low^/CD24^−/low^ cells from luminal cell lines compared to CD44^high^/CD24^−/low^ cells from Hs578T. However, CD44^high^/CD24^−/low^ cells from MDA-MB-231 too showed high expression of NANOG and SOX2. Conversely, FOSL1 and VIM, which are ubiquitously expressed in normal mesenchymal cells and overexpressed in many cancers [37], had a more than 5- to 10-fold higher expression in CD44^high^/CD24^−/low^ cells from triple-negative/basal-like cell lines. However, compared to CD44^−/low^/CD24^−/low^ cells from luminal cell lines, CD44^−/low^/CD24^−/low^ cells from HER2-OE cell line AU-565 had a comparatively lower expression of pluripotency markers but a 2-fold higher expression of SNAIL1, indicating its divergence from luminal cell lines. Surprisingly, within triple-negative cell lines, the proliferation marker MKI67 was highly expressed in CD44^high^/CD24^−/low^ cells from MDA-MB-231 (4-fold higher than that of Hs578T). CD44^−/low^/CD24^−/low^ cells from luminal cell lines overall had a 2- to 3-fold higher expression of CCNA2 and MKI67 than that of CD44^high^/CD24^−/low^ cells from Hs578T (Supplementary Figure S3A). Of all the subpopulations, CD44^−/low^/CD24^−/low^ cells from AU-565 had the lowest expression of both proliferation and pluripotency markers. On the other hand, compared to CD44^high^/CD24^−/low^ cells from Hs578T, CD44^high^/CD24^−/low^ cells from MDA-MB-231 had a higher expression of both pluripotency and proliferation markers.

Our mRNA expression results showed that the CD44^−/low^/CD24^−/low^ subpopulation from luminal cell lines and CD44^high^/CD24^−/low^ subpopulation from highly tumorigenic cell line MDA-MB-231 had similar expression of pluripotency markers and proliferation marker MKI67. We further compared the protein expression of NANOG and MKI67 in mammospheres generated by CD44^−/low^/CD24^−/low^ cells from luminal cell lines with that of mammospheres from CD44^high^/CD24^−/low^ cells of MDA-MB-231. Mammospheres of CD44^−/low^/CD24^−/low^ origin from all luminal cell lines expressed NANOG equivalent to or higher than mammospheres of CD44^high^/CD24^−/low^ origin. BT-474 mammospheres showed significantly higher expression than MDA-MB-231 (*p* < 0.01) (Figure 3D,E). Except for T47D, MKI67 expression in luminal mammospheres was similar to mammospheres from MAD-MB-231 (Figure 3B,C). These results demonstrated that the CD44^−/low^/CD24^−/low^ subpopulation in luminal cell lines may indeed be comprised of cells with pluripotent potential. On the other hand, the overall low expression of proliferation and pluripotency markers in CD44^high^/CD24^−/low^ cells from the Hs578T cell line revealed a stark difference in the expression of these markers within triple-negative cell lines. The overexpression of mesenchymal markers such as vimentin correlated more positively with the CD44^high^/CD24^−/low^ phenotype in triple-negative cell lines.

### 2.4. ALDH1A3 Is Overexpressed in Breast Cancer Subtypes

**Figure 4:**
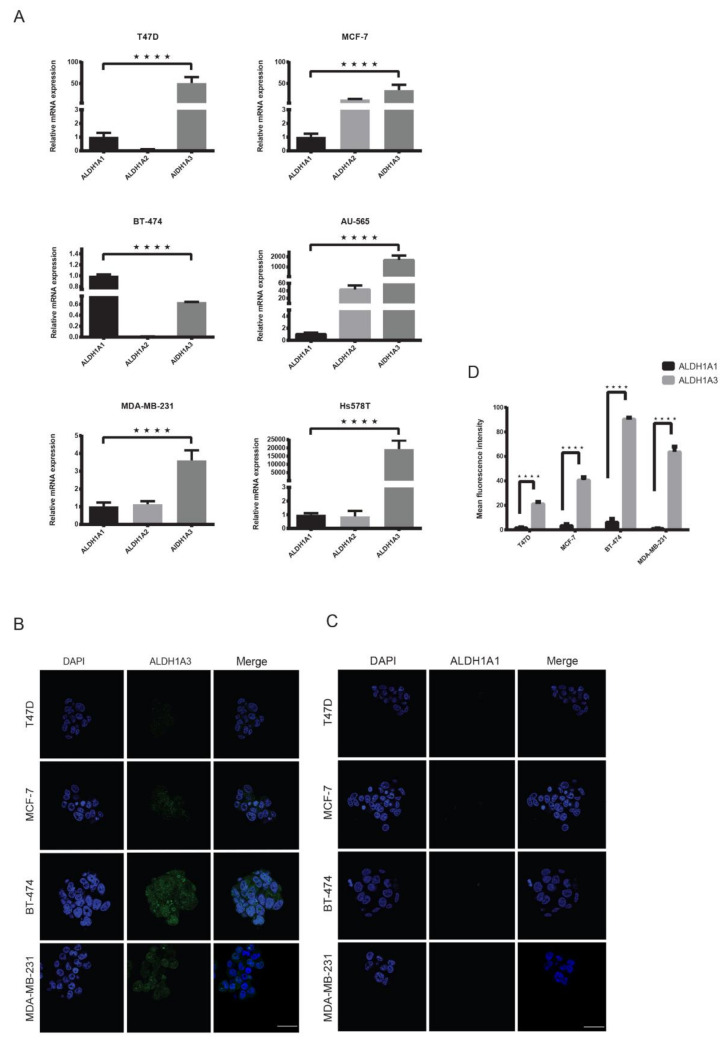
High expression of ALDH1A3 in breast cancer cell lines. (**A**) Relative mRNA expression analysis of ALDH1A1, ALDH1A2 and ALDH1A3 in breast cancer cell lines using qRT-PCR showing a significantly higher expression of ALDH1A3 in all cell lines except BT-474. mRNA expression was normalized to ALDH1A1. (**B**,**C**) Representative image and analysis of immunofluorescence for ALDH1A3 and ALDH1A1 expression in mammospheres showing a significantly higher expression of ALDH1A3. Mammospheres formed after 7 days were separately stained with DAPI (blue), anti-ALDH1A1 (green) and anti-ALDH1A3 (green) antibodies. (**D**) Comparison of A1 and A3 immunofluorescence. Scale bar = 100 μm. Data represent the mean ± SD of three independent experiments; **** *p* < 0.0001.

It has been hypothesized that the ALDH1 isoform ALDH1A1 is responsible for the metastatic property in breast cancer cells with high ALDH activity [25,38]. However, its role as a potential CSC remains controversial [12,22]. In our mammosphere experiments, ALDH^br^ cells did not show high viability and sphere-forming ability. Therefore, to verify this hypothesis, we first compared the expression of ALDH1 isoforms (A1, A2 and A3) in all breast cancer cell lines using qRT-PCR. We found that with the exception of BT-474, ALDH1A1 expression was very low in all cell lines, whereas ALDH1A3 was overexpressed in all lines (*p* < 0.0001) (Figure 4A). To further verify whether the mRNA expression translates to protein expression, we performed an immunofluorescence analysis of ALDH1A1 and ALDH1A3 in long-term mammospheres of luminal cell lines and compared this with the highly tumorigenic MDA-MB-231 cell line. The results showed high ALDH1A3 expression in all cell lines including BT-474 (*p* < 0.0001) (Figure 4B–D), which is surprising as it showed a higher expression of ALDH1A1 in qRT-PCR experiments. Finally, to analyze the claim that ALDH^br^ cells are key tumorigenic cells in HER2-OE breast cancer [14,26], we compared the mRNA expression of the aforementioned key markers genes for pluripotency, proliferation and EMT/metastasis between the CD44^−/low^/CD24^−/low^ and ALDH^br^ subpopulations from the HER2-OE cell lines BT-474 and AU-565. The expression of these genes did not differ significantly between the CD44^−/low^/CD24^−/low^ and ALDH^br^ subpopulations. Overall, these markers were marginally overexpressed in the CD44^−/low^/CD24^−/low^ subpopulation, showing that ALDH^br^ cells may not correlate with EMT, pluripotency or proliferation in HER2-OE cell lines. (Supplementary Figure S3B). Our results support previous studies, which have shown that ALDH1A3 is highly expressed in ALDH^br^ cells and that ALDH1A1 expression may not be directly related to proliferation and metastasis in breast cancer [23,39].

### 2.5. Luminal CD44^−/low^/CD24^−/low^ Cells Show Strong Tumorigenic and Metastatic Behavior

**Figure 5:**
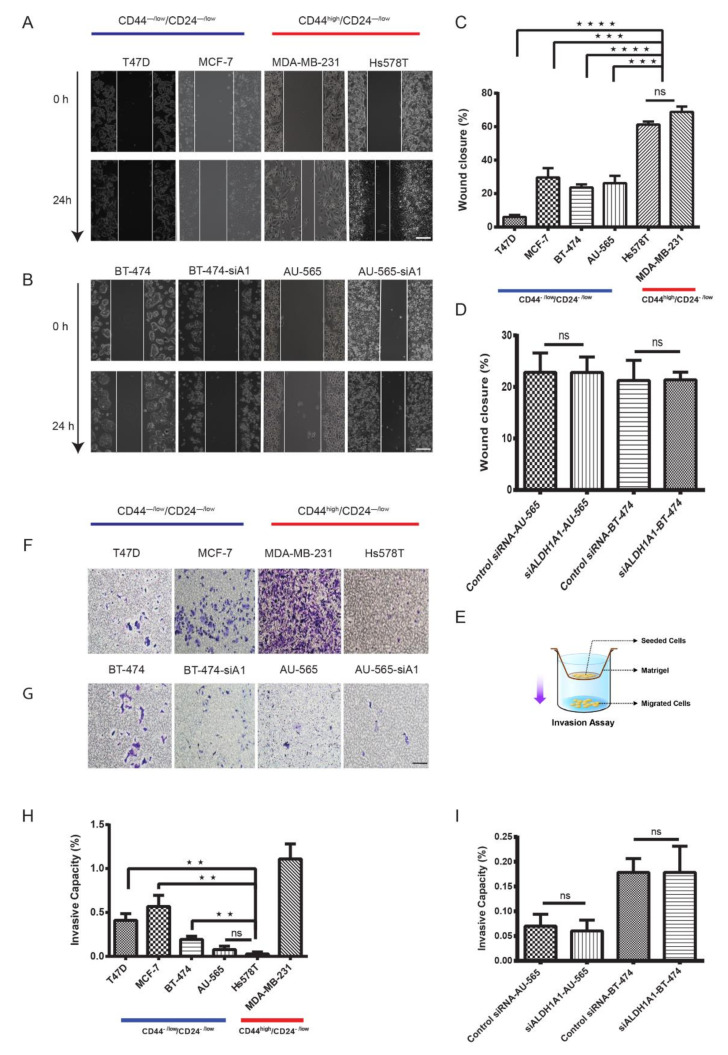
Luminal CD44^−/low^/CD24^−/low^ cells show low migratory but high invasive properties. Representative images and (**A**,**B**) comparison of the wound closure rate (**C**) of different populations show that CD44^high^/CD24^−/low^ cells from triple-negative cell lines had a high rate of cell migration. (**E**) Schematic representation of the transwell invasion assay. The 1 × 10^4^ cells in serum-free medium were seeded in matrigel. Cells migrating to lower chambers filled with complete medium were counted after 24 h under a microscope. (**F**,**G**) Representative images of migrated cells. (**H**) Comparison of invasive capacity showing low invasive properties of CD44^high^/CD24^−/low^ cells and CD44^−/low^/CD24^−/low^ cells from the Hs578T and AU-565 cell lines, respectively. (**D**,**I**) Comparison of siALDH1A1 with control in HER2-OE cell lines for wound healing and transwell invasion assays. Data represent the mean ± SD (n = 3); ** *p* < 0.01, *** *p* < 0.001, and **** *p* < 0.0001. Scale bar = 200 μm.

The ability of cancer cells to proliferate, migrate and invade other tissues is a key aspect of metastasis which leads to mortality in cancer patients [40]. To investigate migratory and invasive properties in the CD44^−/low^/CD24^−/low^ subpopulation, we performed transwell invasion and wound healing assays. To further assess the metastatic potential of these cells, we performed xenotransplantation of CD44^−/low^/CD24^−/low^ cells into the mammary fat pad of 6-week-old female NOD-*scid IL2r*γ (NSG) mice. Expectedly, the CD44^high^/CD24^−/low^ subpopulation from the highly invasive MDA-MB-231 cell line had the highest number of cells with random migration/invasion. However, CD44^−/low^/CD24^−/low^ cells from luminal cell lines MCF-7, T47D and BT-474 had a significantly higher number of (3–5 times) invasive cells than the CD44^high^/CD24^−/low^ subpopulation from Hs578T and the CD44^−/low^/CD24^−/low^ subpopulation from AU-565 (Figure 5F–H). The wound healing results, however, did not mirror those of the transwell assay—CD44^high^/CD24^−/low^ cells from the triple-negative cell lines MDA-MB-231 and Hs578T had the highest rate of migration over a period of 24 h, whereas CD44^−/low^/CD24^−/low^ cells from luminal cell lines had a significantly lower rate of migration (Figure 5A–C).

Our analysis of ALDH1 isotypes showed a low expression of ALDH1A1 in breast cancer cell lines. This led us to suspect that ALDH1A1, which is believed to be the key promoter of tumorigenicity in HER2-OE breast cancer [14,26], may not play a key role in tumorigenicity. We knocked down ALDH1A1 using siRNA in HER2-OE cell lines BT-474 and AU-565 and further compared its effect with normal cells in both the AU-565 and BT-474 cell lines. Transwell invasion and wound healing assays show that, as suspected, the suppression of ALDH1A1 activity in these two cell lines did not significantly affect their proliferation or migration (Figure 5D,I).

**Figure 6:**
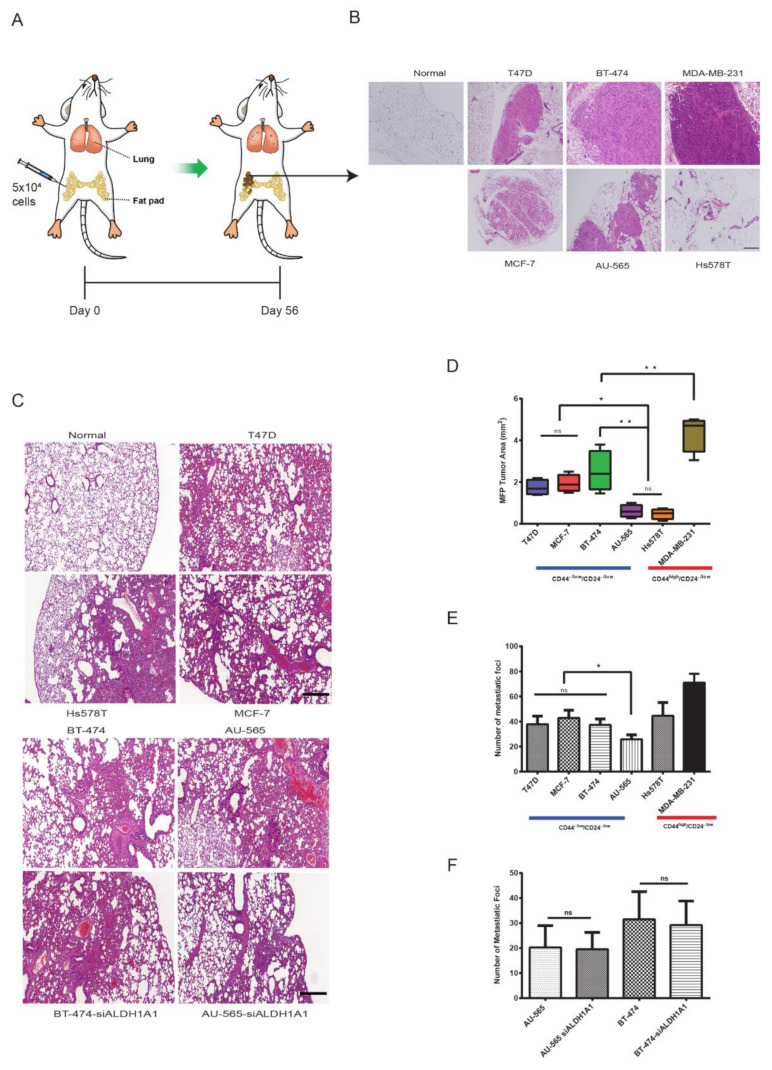
Luminal CD44^−/low^/CD24^−/low^ cells cause metastasis in NSG mice. (**A**) Schematic representation of the xenotransplant experiment. Female NSG mice were injected with 5 × 10^4^ cells from CD44^high^/CD24^−/low^, CD44^−/low^/CD24^−/low^ and siALDH1 populations. The mammary fat pad (MFP) and lungs were compared for tumorigenic and metastatic potential after 8 weeks of incubation. (**B**) Representative images of H&E staining of the site of injection in the MFP. (**C**) Representative images of H&E staining of lung metastasis. (**D**) Analysis of tumor area in the MFP of NSG mice. (**E**) Analysis of lung tumor foci. (**F**) Comparison of lung tumor metastatic foci between non-si and siALDH1A1 in HER2-OE cell lines. Data represent the mean ± SD of three independent experiments; * *p* < 0.05 and ** *p* < 0.01. Scale bar = 200 μm.

Finally, we compared the tumorigenic and metastatic potential of these populations in xenotransplanted 6-week-old female NSG mice. FACS-isolated 5 × 10^4^ cells were injected in the mammary fat pad (MFP) at the 4th or 5th nipple site orthotopically and, after 56 days (8 weeks), lungs and the MFP were harvested. Locally, at the site of injection, MDA-MB-231 CD44^high^/CD24^−/low^ xenotransplants formed the largest tumors. However, luminal CD44^−/low^/CD24^−/low^ xenotransplants formed larger tumors than Hs578T (*p* < 0.05) (Figure 6B,D). In terms of lung metastasis, CD44^−/low^/CD24^−/low^ xenotransplants from luminal cell lines formed significantly more metastatic foci (≥200 µm) compared to CD44^−/low^/CD24^−/low^ xenotransplants from AU-565 (*p* < 0.05). CD44^high^/CD24^−/low^ xenotransplants from the triple-negative MDA-MB-231 cell line showed a higher number of metastatic foci compared to CD44^−/low^/CD24^−/low^ xenotransplants from luminal cell lines, while CD44^high^/CD24^−/low^ xenotransplants from Hs578T had only a marginally higher number of metastatic foci compared to CD44^−/low^/CD24^−/low^ xenotransplants from the luminal cell lines T47D and BT-474 (Figure 6C,E). Knockdown of ALDH1A1 did not affect the metastatic efficiency of ALDH^br^ cells in HER2-OE cell lines (Figure 6C,F).

**Figure 7:**
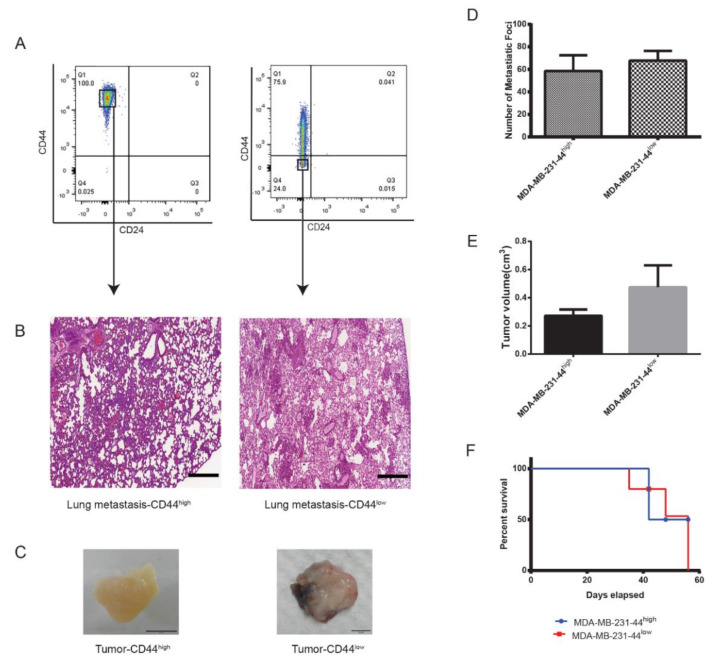
High tumorigenicity in MDA-MB-231 is not CD44 dependent. Analysis of the CD44^high^/CD24^−/low^ and CD44^−/low^/CD24^−/low^ populations in MDA-MB-231. Cells from short-term (7-days) and long-term (21 days) mammosphere culture were isolated by FACS (**A**) and injected in the MFP of female NSG mice as described earlier. (**B**) Representative images of H&E-stained lung metastasis. (**C**) Representative images of solid tumors. Scale bar = 0.5 cm. Comparison of lung metastatic foci (**D**), tumor volume (**E**), and survival rate (**F**). Data represent the mean ± SD of three independent experiments. Scale bar = 200 μm.

The large difference in tumorigenic behavior within triple-negative cell lines Hs578T and MDA-MB-231 led us to speculate that CD44^high^/CD24^−/low^ cells may not always correlate to highly tumorigenic and invasive behavior in triple-negative cell lines. To further confirm this, we xenotransplanted 1000 cells of the CD44^−/low^/CD24^−/low^ phenotype isolated from the mammosphere of MDA-MB-231 in the mammary fat pad of NSG mice. Both the CD44^high^/CD24^−/low^ and CD44^−/low^/CD24^−/low^ populations had comparable metastatic efficiency with the CD44^−/low^ population forming slightly larger tumors (Figure 7C–E). Further, mice injected with the CD44^−/low^/CD24^−/low^ population showed higher mortality than mice injected with CD44^high^/CD24^−/low^ cells (Figure 7G). Overall, MDA-MB-231-derived CD44^−/low^/CD24^−/low^ cells showed high malignancy, forming a large number of lung metastatic foci, and shortened disease-free survival.

These results demonstrated that, while CD44^high^/CD24^–/low^ cells from both the triple-negative cell lines had a significantly higher migration rate than CD44^−/low^/CD24^−/low^ cells from luminal cell lines, the CD44^high^/CD24^−/low^ cells from Hs578T were only weakly invasive, less so than CD44^−/low^/CD24^−/low^ cells from luminal cell lines. ALDH^br^ cells did not show a significant difference in proliferative or migratory potential from CD44^−/low^/CD24^−/low^ cells in their respective cell lines. Consistently, the mouse xenotransplant study revealed a similar pattern, which showed that CD44^−/low^/CD24^−/low^ xenotransplants from luminal cell lines had higher tumorigenicity than CD44^−/low^/CD24^−/low^ xenotransplants from AU-565 and CD44^high^/CD24^−/low^ xenotransplants from Hs578T. However, in terms of lung metastasis, only CD44^−/low^/CD24^−/low^ xenotransplants from AU-565 showed significantly lower metastasis than CD44^−/low^/CD24^−/low^ xenotransplants from luminal cell lines. The overall weak tumorigenic and metastatic behavior of the AU-565 subpopulations is in keeping with the in vitro and gene expression results. CD44^high^/CD24^−/low^ xenotransplants from the MDA-MB-231 cell line, had the highest tumorigenicity as well as metastatic potential. However, within the MAD-MB-231 cell line, mammosphere-isolated CD44^−/low^/CD24^−/low^ xenotransplants showed marginally higher tumorigenicity and metastatic potential than CD44^high^/CD24^−/low^ xenotransplants. Although various reports have concluded that CD44 expression is critically associated with high tumorigenicity, a direct correlation is lacking [41]. In our experiments, the CD44^high^/CD24^−/low^ phenotype in triple-negative cell lines seems to be positively correlated with proliferation and migration. Moreover, CD44^−/low^/CD24^−/low^ cells from luminal cell lines show low proliferative capacity but high tumorigenic and metastatic properties. Overall, CD44^−/low^/CD24^−/low^ cells from luminal breast cancer cell lines seem to behave akin to pluripotent quiescent, slow-cycling cells.

### 2.6. CD44^−/low^/CD24^−/low^ Xenotransplants Express NANOG and ALDH1A3 at Metastatic Site

**Figure 8:**
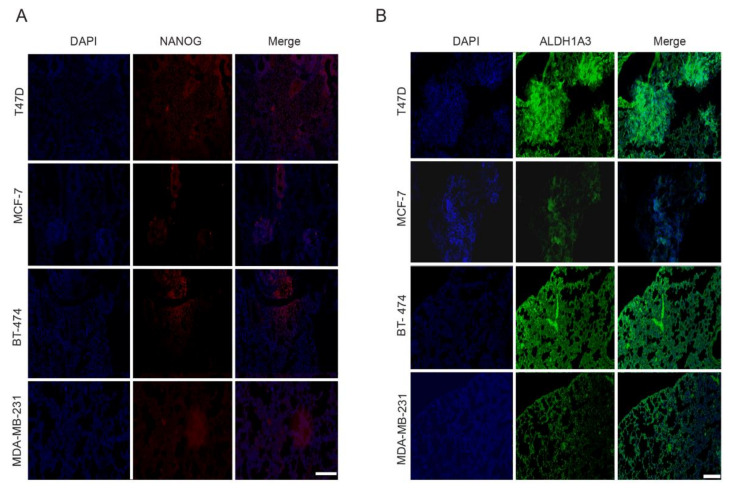
Luminal CD44^−/low^/CD24^−/low^ cells express NANOG and ALDH1A3 at the metastasis site. Representative images of double immunohistostained metastatic sections of lungs from NSG mice. (**A**) DAPI (blue), anti-NANOG (red) (**B**) DAPI (blue), anti-ALDH1A3 (green). Scale bar = 200 μm.

There is increasing evidence that the gene expression profile of CSCs or tumorigenic cells depends on the changes in their microenvironment and thus is dynamic [42,43]. This aspect may complicate the identification of CSC-specific biomarkers. This dynamism would be reflected in the differential gene expression observed from the primary tumor site to the site of metastasis in CSCs. To assess this dynamic, we analyzed the expression of NANOG and ALDH1A3 in lung metastatic sites by immunohistofluorescence. These genes are highly expressed in luminal CD44^−/low^/CD24^−/low^ and MDA-MB-231 cells, as demonstrated by our qRT-PCR and immunofluorescence analysis. Our analysis shows that both NANOG and ALDH1A3 were consistently expressed at the metastatic site (Figure 8A,B) in all luminal xenotransplants. Mice xenotransplanted with CD44^high^/CD24^−/low^ cells from the triple-negative MDA-MB-231 cell line showed a similar expression of both NANOG and ALDH1A3. Overall, our results show that the expression of NANOG and ALDH1A3 remains stable during metastasis, probably not affected by change in the tumor microenvironment.

## 3. Discussion

Identification of tumorigenic cells specific for luminal breast cancer, which represents more than 70% of all breast cancers, will significantly improve understanding of tumor microenvironment and signaling events that are involved in discrete steps of breast cancer progression, including metastasis. This insight is critical because, despite improved prognosis of breast cancer due to the availability of targeted agents, drug resistance and tumor recurrence still remain major concerns. Here, we show that, in luminal breast cancer cell lines, cells with the CD44^−/low^/CD24^−/low^ phenotype display features typically associated with cells with tumorigenic and metastatic property.

Previous studies have shown cells with the CD44^high^/CD24^−/low^ and ALDH^br^ phenotypes to be highly tumorigenic, potential BCSCs [12,24,25,44,45]. Cells with high ALDH activity are considered to possess stem cell-like properties in both normal and malignant cells [13,46] and ALDH1A1 expression has been shown to correlate with higher metastasis and worse prognosis in clinical samples of breast cancer [23,47]. However, a direct association between these phenotypes and invasion, homing and proliferation at sites of metastasis is lacking [21]. MCF-7, which is a luminal A, ER-positive cell line, is one such example to the contrary. The CD44^−/low^/CD24^−/low^ subpopulation from MCF-7 causes osteosclerotic bone lesions on intracardiac injection in nude mice, showing high invasiveness and metastatic potential [48]. By comparing and analyzing the tumorigenic and metastatic properties of these two phenotypes with the CD44^−/low^/CD24^−/low^ phenotype, we found that tumorigenic cells in luminal cell lines typically had a CD44^−/low^/CD24^−/low^ phenotype, whereas the CD44^high^/CD24^−/low^ phenotype was associated with triple-negative/basal-like cell lines. Similar to previous studies [29], we found that ALDH expression was the strongest in HER2-OE cell lines, while it was very low in luminal and triple-negative/basal-like cell lines. Contrary to previous reports, we did not find differences in the tumorigenic ability of ALDH^br^ cells vis-a-vis CD44^−/low^/CD24^−/low^ cells. In terms of invasiveness, metastasis and tumorigenicity, CD44^−/low^/CD24^−/low^ cells from MDA-MB-231 had the highest tumorigenic and invasive capacity, whereas these properties were the weakest in CD44^high^/CD24^−/low^ and ALDH^br^ cells from the Hs578T and AU-565 cell lines, respectively. These results suggest that the CD44^high^/CD24^−/low^ and ALDH^br^ phenotypes may not universally relate to high tumorigenicity or invasiveness in breast cancer subtypes. Our results are more supportive of the argument put forth in a few previous studies [49,50] that these phenotypes could be markers for HER2-OE and triple-negative/basal-like breast cancer.

The role of ALDH isozyme ALDH1A1 in cancer has been a subject of intensive study, with a number of reports implicating that it is a key factor in maintaining stem cell-like properties in ALDH^br^ cells while some reports dispute this role [22,51,52]. We therefore determined the role of ALDH1A1 in ALDH^br^ cells by first measuring its mRNA and protein expression and then using siRNA to specifically knockdown ALDH1A1. At the mRNA level, except for BT-474, all cell lines showed a significantly high level of ALDH1A3 but not A1. Moreover, knockdown of ALDH1A1 did not show a significant difference in proliferation, migration or metastasis in ALDH^br^ cell lines. The CD44^−/low^/CD24^−/low^ subpopulation showed self^−^renewal, proliferative and metastatic properties equivalent to the ALDH^br^ subpopulation in HER2-OE cell lines. Interestingly, ALDH^br^ cells from luminal A subtype did not show tumorigenic and metastatic properties and did not survive long-term sphere culture, indicating that ALDH activity may not a key marker for cancer progression in luminal A cell lines. Our results also show that ALDH1A3 is overexpressed in breast cancer cell lines of different subtypes. Further studies are required to ascertain whether the overexpression of ALDH1A3 plays a role in tumorigenesis and metastasis. Overall, our results show that ALDH1A1 expression is not consistent across breast cancer subtypes, and the presence of ALDH^br^ cells may not be a marker of strong pluripotency or metastatic behavior. The presence and proportion of ALDH^br^ cells in MDA-MB-231 is unclear. Some investigators have reported no presence of ALDH^br^ cells in MDA-MB-231, whereas others have reported the presence of a small CD44^high^/CD24^−/^ALDH^br^ subpopulation considered to be highly tumorigenic [17,21,25,26]. However, in our experiments, we could not find ALDH^br^ cells in the MDA-MB-231 cell line. We were not able ascertain whether CD44^high^/CD24^−/low^/ALDH^br^ cells did indeed have a comparatively higher tumorigenic potential than CD44^high^/CD24^−/low^ cells. A more detailed study encompassing an in^-^depth mechanistic and transcriptomic experimental design is required in order to understand the role of ALDH^br^ cells in breast cancer.

Gene expression analysis of key EMT, metastasis and pluripotency markers has been widely implied in many studies to identify tumorigenic or stem cell-like cells in cancer [53]. Our qRT-PCR and immunofluorescence analysis of a number of these genes in CD44^high^/CD24^−/low^, CD44^−/low^/CD24^−/low^ and ALDH^br^ cells shows that luminal CD44^−/low^/CD24^−/low^ cells and CD44^high^/CD24^−/low^ cells from the triple-negative MDA-MB-231 cell line overexpress stem cell marker genes *NANOG, KLF4* and *SOX2* compared to their counterparts from AU-565 and Hs578T—both of which showed significantly low invasiveness in the transwell assay. *SNAIL2, VIM* and *FOSL1* genes, which are typically associated with EMT/metastasis and mesenchymal cells [54], had a higher expression in both the triple-negative cell lines. ALDH^br^ cells from both BT-474 and AU-565 cell lines showed expression profiles very similar to CD44^−/low^/CD24^−/low^ cells of their respective cell lines. Expression analysis of proliferation marker MKI67 showed that except for T47D, MKI67 was overexpressed in CD44^−/low^/CD24^−/low^ cells from luminal and CD44^high^/CD24^−/low^ cells from MDA-MB-231 cell lines. The sharp difference in the invasive and metastatic behavior and expression profile of key pluripotency markers between CD44^high^/CD24^−/low^ cells from the Hs578T and MDA-MB-231 cell lines does indicate an intrinsic variation in tumorigenic potential within cell lines with CD44^high^/CD24^−/low^ phenotype. It would seem that the relatively high expression of pluripotency makers in luminal CD44^−/low^/CD24^−/low^ cells is the reason for their long-term survival and tumorigenicity, perhaps also contributing to therapy resistance [55]. Further investigation of the tumorigenic potential of CD44^−/low^/CD24^−/low^ cells in luminal breast cancer will be the key to understanding their tumorigenic potential and in developing biomarkers which identify the tumorigenic cells with more accuracy.

The importance of NANOG in the maintenance of pluripotency and proliferation is well documented [56,57]. It is therefore likely that upregulation of NANOG expression is critically related to tumorigenic cells in cancer [58]. Recently, Lu et al. [35] elucidated the role of NANOG in promoting breast cancer metastasis. Our comparison of NANOG expression in CD44^−/low^/CD24^−/low^ cells from luminal cell lines and CD44^high^/CD24^−/low^ cells from the highly invasive triple-negative MDA-MB-231 cell line showed similar expression levels, indicating their potential key role in maintaining pluripotency in these cell populations. Aberrations in developmentally conserved signaling pathways such as Janus kinase (JAK)/signal transducer activator of transcription protein (STAT) and activator of transcription 3 (STAT3) signaling pathways are deemed to be critical for the formation of CSCs [59]. In the JAK/STAT pathway, the phosphorylation of *STAT3* results in enhancement of stem cell marker genes *POU5F1, SOX2, KLF4* and *NANOG*, leading to tumorsphere formation and drug resistance [60,61]. It is therefore likely that the activation of the JAK/STAT pathway is the key factor for the tumorigenic behavior of CD44^−/low^/CD24^−/low^ cells. Further, various other signaling pathways and signaling crosstalk could contribute to the formation of breast CSCs, and therefore further research into the activation of these signaling pathways, particularly the JAK/STAT pathway, is required in order to understand the genetic and molecular mechanism involved in the maintenance of tumorigenic populations in breast cancer.

As our results are based on representative cell lines, further research with primary and metastatic tumor-derived samples from both luminal and triple-negative breast cancer patients representing different age groups is critical in order to understand the tumorigenic role of these phenotypes in breast cancer.

## 4. Materials and Methods

### 4.1. Antibodies and Reagents

Supplementary Table S1 provides a list of all reagents and antibodies used.

### 4.2. Cell Culture

All cell lines were obtained from the Bioresource Collection and Research Center, Taipei, Taiwan. T47D, AU-565, MCF-7, BT-474, Hs578T and MDA-MB-231 cells were cultured in Dulbecco’s Modified Eagle Medium (DMEM) supplemented with 10% fetal bovine serum (FBS) and 1% penicillin/streptomycin. Cells were maintained in a humidified atmosphere of 5% CO_2_/95% air at 37 °C. Cells were subcultivated using 0.25% trypsin and 5 mM Ethylenediaminetetraacetic acid (EDTA).

### 4.3. Mammosphere Culture

Cell lines were plated into ultra-low-attachment plates (Corning, NY, USA) at a density of 2 × 10^4^ cells/mL and cultured in serum-free DMEM/F12 (1:1) medium supplemented with 20 ng/mL epidermal growth factor (EGF, PeproTech, St. Louis, MO, USA), 10 ng/mL basic fibroblast growth factor (b-FGF, PeproTech, St. Louis, MO, USA), ITS (insulin, transferrin and selenium, Sigma-Aldrich, St. Louis, MO, USA), and B27 (GIBCO, Waltham, MA, USA). A volume of 2 mL fresh mammosphere media was added every 2 to 3 days without decanting old media. Mammospheres were either disassociated using Accutase reagent or collected every 7, 14, 21 and 42 days for further analysis. Mammosphere-forming efficiency (MFE) was calculated using the formula: MFE = (number of mammospheres counted/number of cells plated) × 100.

### 4.4. ALDEFLUOR Assay

Breast cancer cells at the logarithmic growth phase were digested with 0.25% trypsin or Accutase (from Mammosphere) and washed three times with phosphate-buffered saline (PBS), followed by resuspension in 100 µl PBS. The ALDEFLUOR kit (StemCell Technologies, Durham, NC, USA) was used to analyze the population with high ALDH enzymatic activity. Briefly, the cells were incubated in the ALDEFLUOR assay buffer containing ALDH substrate BODIPY-aminoacetaldehyde (BAAA), 1 μmol per 1 × 10^6^ cells, and incubated for 40 min at 37 °C. Each experiment included a negative control that contained 50 mM diethylaminobenzaldehyde (DEAB), which specifically inhibits ALDH activity.

### 4.5. Fluorescence-Activated Cell Sorting (FACS)

Immediately after ALDEFLOUR staining, the cells were stained with anti-CD44-APC (allophycocyanin) and anti-CD24-PerCP-eFluor 710, and separate aliquots of cells were single stained with anti-CD44-APC and anti-CD24-PerCP-eFluor 710 and used as controls. Aliquots were also treated with their isotype controls at 4 °C in the dark for 15 min. Dead cells were stained with 7-aminoactinomycin D (7-AAD). The samples were then washed by PBS three times and finally re-suspended in 500 μL PBS. Flow cytometry analysis was performed with a BD Facs Aria IIIu Flow Cytometer (BD Bioscience Franklin Lakes, NJ, USA).

### 4.6. Colony Formation Assay

After flow cytometry, separated cells were directly seeded on 24-well plates (Corning, NY, USA, #3526) supplemented with DMEM containing 10% fetal bovine serum. Cells were plated at different densities (100, 500 and 1000 cells/well) with 3 replicates of each density, and the plates were incubated at 37 °C in a 5% CO_2_ environment for 1–3 weeks depending on the cell line. The plates were then washed with 0.9% saline and fixed with 10% neutral buffered formalin solution for 15–30 min. The wells of each plate were stained with 5 mL 0.01% (w/v) crystal violet in dH_2_O for 30−60 min, washed with distilled water and counted using a microscope or stereomicroscope. Colonies containing more than 50 individual cells were counted and image captured. Colony forming efficiency (CFE) was calculated using the formula: CFE = (number of colonies counted/number of cells plated) × 100.

### 4.7. Transwell Migration Assay

The Transwell assay was applied using the transwell chamber with 8.0 μm pore polycarbonate membrane inserts (Corning, NY, USA, #3422). Flow cytometry isolated subpopulations from the aforementioned breast cancer cell lines were seeded into the upper chambers of the inserts (1 × 10^4^ cells/chamber) in 200 μL serum-free DMEM/F-12 medium at 37  °C. Complete DMEM (600 μL) was added in the lower chambers. After 48 h of incubation, cells migrating to lower chambers were dyed with crystal violet and counted under a microscope.

### 4.8. Wound Healing Assay

FACS-sorted or siRNA-treated cells were plated onto a 6-well plate to create confluent monolayers. Then 2-well culture inserts (Ibidi, Martinsried, Planegg, Germany #80209) were used in petri plates (Corning, NY, USA, #353801) to create a uniform “scratch” area across each monolayer. The insert was removed, and 2 mL complete medium was added. The dishes were placed under a live cell-imaging phase-contrast microscope (Leica DMI 6000 B, Wetzlar, Germany) and images were acquired at 1 h intervals. For each image, the distance between either side of the scratch was measured at certain intervals (mm). By comparing the distances from time 0 to the last time point (24 h), the migration distance of each cell was obtained.

### 4.9. Immunofluorescence, Immunohistochemistry, and Image Quantitative Analysis

For monolayers, cells were plated at a concentration of 5 × 10^4^ cells/mL in the wells of 8-well chamber slides (Ibidi Martinsried, Planegg, Germany). The cells were fixed with 4% PFA for 20 min and permeabilized with PBS containing 0.2% (w/v) Triton X-100 on the following day. The cells were then blocked for 30 min at 37 °C with 5% bovine serum albumin (BSA) and incubated at 37 °C for 1 h with anti-CD44 primary antibody and anti-CD24-PE. Mammospheres acquired after 7 days in culture were incubated at 37 °C for 1 h with unconjugated primary antibodies for ALDH1A1, ALDH1A3, and NANOG and MKI67 (conjugated with Alexa Fluor 647). Cells stained with unconjugated primary antibodies were then washed with PBS and further stained for 1 h in dark at 37 °C. Cells incubated with primary antibodies were further stained with the appropriate secondary antibodies. Nuclei were counterstained with 4′,6-diamidino-2-phenylindole (DAPI, Invitrogen, Waltham, MA, USA). The samples were then washed twice with PBS and mounted with anti-bleaching coverslips. For histochemical staining, tissue sections were stained with hematoxylin and eosin (H&E). For immunohistostaining, Paraffin-embedded sections were deparaffinized in xylene and rehydrated in a graded series of ethanol. Antigen enhancement was carried out by incubating the sections in citrate buffer, pH 6 (Dakocytomation, Copenhagen, Denmark). Double fluorescence staining for ALDH1A3 and NANOG was carried out using the aforementioned procedure. All the samples were examined and photographed using a single-photon laser confocal imaging system, Zeiss510 (Carl Zeiss, Oberkochen, Baden-Württemberg, Germany).

### 4.10. Mouse Xenotransplant

All the animal experiments were performed according to guidelines for the care and use of laboratory animals of the Animal Study Committee of the Institute of Biomedical Sciences, Academia Sinica, and were carried out according to the protocol approved by the Institutional Animal Care Facility (IACUC-14-9-733). Mice were maintained under specific pathogen-free conditions, and all the efforts were made to minimize animal suffering. Female NOD-*scid IL2r*γ (NSG) mice at 6 weeks of age were purchased from Animal Laboratory, Institute of Biomedical Sciences, Academia Sinica. After trypsinization, different subpopulations from breast cancer cell lines were isolated by flow cytometry. The cells were washed once with PBS, resuspended in culture medium at 5 × 10^4^ cells per 100 µL, and injected in triplicate into mammary fat pads of female NSG mice. Mice with xenotransplants from ER+ cell lines (T47D, MCF-7 and BT-474) were supplemented with 0.18 mg 60-day release 17β-estradiol (Innovative Research of America, Sarasota, FL, USA, SE-121). The mice were monitored daily for 8 weeks. In the case of palpable tumors, size was measured with calipers after sacrifice. The tumor volume was determined by the formula: tumor volume = 1/2(length × width2). Lungs, the mammary fat pad and solid tissue (if applicable) were obtained from mice after euthanization, fixed in 4% paraformaldehyde (PFA) (Sigma, St. Louis, MO, USA) at 4 °C and processed for H&E and immunohistochemical staining by embedding in paraffin. Average lung metastatic foci were estimated by counting the number of metastases of size 200 µm or greater in each H&E-stained lung section.

### 4.11. Quantitative Reverse Transcription-PCR (qRT-PCR)

Total RNA was extracted according to the RNeasy microextraction kit (Qiagen, Hilden, Germany, #74004) from the FACS-isolated phenotypes. Extracted RNA was reverse-transcribed to generate first-strand cDNA (QuantiTect Reverse Transcription Kit, Qiagen, Hilden, Germany) for use in qPCR. qPCR was performed with Dnase-treated RNA using the Power SYBR Green Master Mix (Thermofisher Scientific, Waltham, MA, USA) on a real-time PCR system (Applied Biosystems, Waltham, MA, USA). qPCR primers were designed using PrimER-Blast software (http://www.ncbi.nlm.nih.gov/tools/primer-blast) and were based on previous studies. Supplementary Table S2 lists all primers used for this study.

### 4.12. RNA Silencing

Double-stranded ALDH1 (SMARTpool: ON-TARGETplus ALDH1 siRNA, L-008722-00-0005 5 nmol) siRNA and a scrambled control siRNA were purchased from Dharmacon (Lafayette, CO, USA). Cells at 50% confluence were transfected with ALDH1A1 siRNA in triplicate in 2 mL complete medium in 6-well plates. Transfections were performed with 50 nM siRNA using transfection reagent (DharmaFECT 1, T-2001-02, Dharmacon). The cells were then incubated at 37 °C in 5% CO_2_ for 48 h, harvested, and processed for further assays.

### 4.13. Statistical Analysis

GraphPad Software (version 8.0.0 for Windows, San Diego, CA, USA) and R package ggplot2 (https://ggplot2.tidyverse.org) were used to analyze data. Two-tailed student’s t-tests and one-way analysis of variance (ANOVA) were used to determine statistical differences. *p*-values <0.05 were considered statistically significant.

## 5. Conclusions

Tumorigenic cells for luminal breast cancers, which represent the majority of breast cancers, remain to be identified. In this report, we show that luminal breast cancer cell lines are enriched in CD44^−/low^/CD24^−/low^ cells, which show characteristic properties of stemness and tumorigenicity, and we therefore conclude that this population contains tumorigenic and/or cancer progenitor cells.

## Supplementary Materials

The following are available online at https://www.mdpi.com/2072-6694/12/5/1239/s1, Figure S1: Gating strategy to isolate ALDH^br^, CD44^high^/CD24^−/low^ and CD44^−/low^/CD24^−/low^ populations, Figure S2: Luminal CD44^−/low^/CD24^−/low^ cells form tightly packed spheres, Figure S3: Relative expression of markers for pluripotency, proliferation and EMT/metastasis, Table S1: Antibodies and reagents used, Table S2: Primers used for qRT-PCR.
