# Supplementary material for: Tumorigenic and Metastatic Role of CD44−/low/CD24−/low Cells in Luminal Breast Cancer
Source: Cancers (Basel). 2020 May 14;12(5):1239. doi: 10.3390/cancers12051239 (PMC7281029; doi:10.3390/cancers12051239)
Supplement: Supplementary file 1 [file cancers-12-01239-s001.pdf]

Table S1: Antibodies and reagents used

Antibody	Company	Catalog No	Host Species
Anti-CD44-APC	Thermofisher	MA1-10226	Mouse
Anti-CD24-PerCP-eFluor 710	Thermofisher	46-0247-42	Mouse
Primary anti-CD44	Abcam	ab157107	Rabbit
Primary anti-CD24	Abcam	ab202073	Rabbit
Primary Anti-ALDH1A3	Abcam	ab129815	Rabbit
Anti-ALDH1A1- Alexa Fluor 488	Abcam	ab195254	Rabbit
Primary Anti-NANOG	Abcam	ab109250	Rabbit
Anti-KI67-Alexa Fluor 647	Abcam	ab196907	Rabbit
Secondary- Goat anti-rabbit FITC	Jackson ImmunoResearch	111-095-003	Goat
Secondary- Goat anti-rabbit TRITC	Jackson ImmunoResearch	111-025-003	Goat
**Reagent**			
Penicillin-Streptomycin,-100X	ThermoFischer	15140-122	
B27 supplement -50x	Gibco	17504-044	
Fibroblast growth factor	Gibco	PHG0314	
Epidermal growth factor	Gibco	PHG0314	
D-MEM/F-12	Gibco	12500-039	

Table S2: Primers used for qRT-PCR

Gene	Transcript	Forward primer	Reverse primer
**EMT/Metastasis**			
SNAIL1	ENSG00000124216	TAATCCAGAGTTTACCTTCCAGCA	AGCCTTTCCCACTGTCCTCA
SNAIL2	ENSG00000019549	GCCAAACTACAGCGAACTGG	AGGAGGTGTCAGATGGAGGA
FOSL1	ENSG00000175592	GCAGGCGGAGACTGACAA	GGGGAAAGGGAGATACAAGG
VIM	ENSG00000026025	CAGATGCGTGAAATGGAAGA	TGGAAGAGGCAGAGAAATCC
CDH2	ENSG00000170558	CATTATCAACCCCATCTCGG	ACTGTCCCATTCCAAACCTG
**Pluripotency**			
POU5F1	ENSG00000204531	CGAAAGAGAAAGCGAACCAG	AACCACACTCGGACCACATC
NANOG	ENSG00000111704	CCTATGCCTGTGATTTGTGG	AAGTGGGTTGTTTGCCTTTG
SOX2	ENSG00000181449	ACACCAATCCCATCCACACT	CCTCCCCAGGTTTTCTCTGT
REX1/ZFP42	ENSG00000179059	CGCGGTAACAGGGACAAATG	TGCAGGTCTTGGCTTGACTT
KLF4	ENSG00000136826	ACCTTCTTCACCCCTAGAGC	CGGTAGTGCCTGGTCAGTTC
**Proliferation**			
CCNA2	ENSG00000145386	AAGACGAGACGGGTTGC	GGCTGTTTACTGTTTGCTTTCC
ERBB2	ENSG00000141736	GGCACAGTCTACAAGGGCA	ATTTCTTTGTTGGCTTTGGG
MKI67	ENSG00000148773	TGGGTCTGTTATTGATGAGCC	CATCAGGGTCAGAAGAGAAGC
**ALDH Isotype**			
ALDH1A2	ENSG00000128918	CCATTGGAGTGTGTGGACAG	GATGAGGGCTCCCATGTAGA
ALDH1A1	ENSG00000165092	GCACGCCAGACTTACCTGTC	CCTCCTCAGTTGCAGGATTAAAG
ALDH1A3	ENSG00000184254	AAAAAGAGCGAATAGCACCG	GCATAGAGGGCGTTGTAGCA
**Surface markers**			
CD44	ENSG00000026508	GAAGAAGGTGTGGGCAGAAGA	ACCATTTCCTGAGACTTGCTG
CD24	ENSG00000272398	GCTCCTACCCACGCAGATT	GCTCCTACCCACGCAGATT

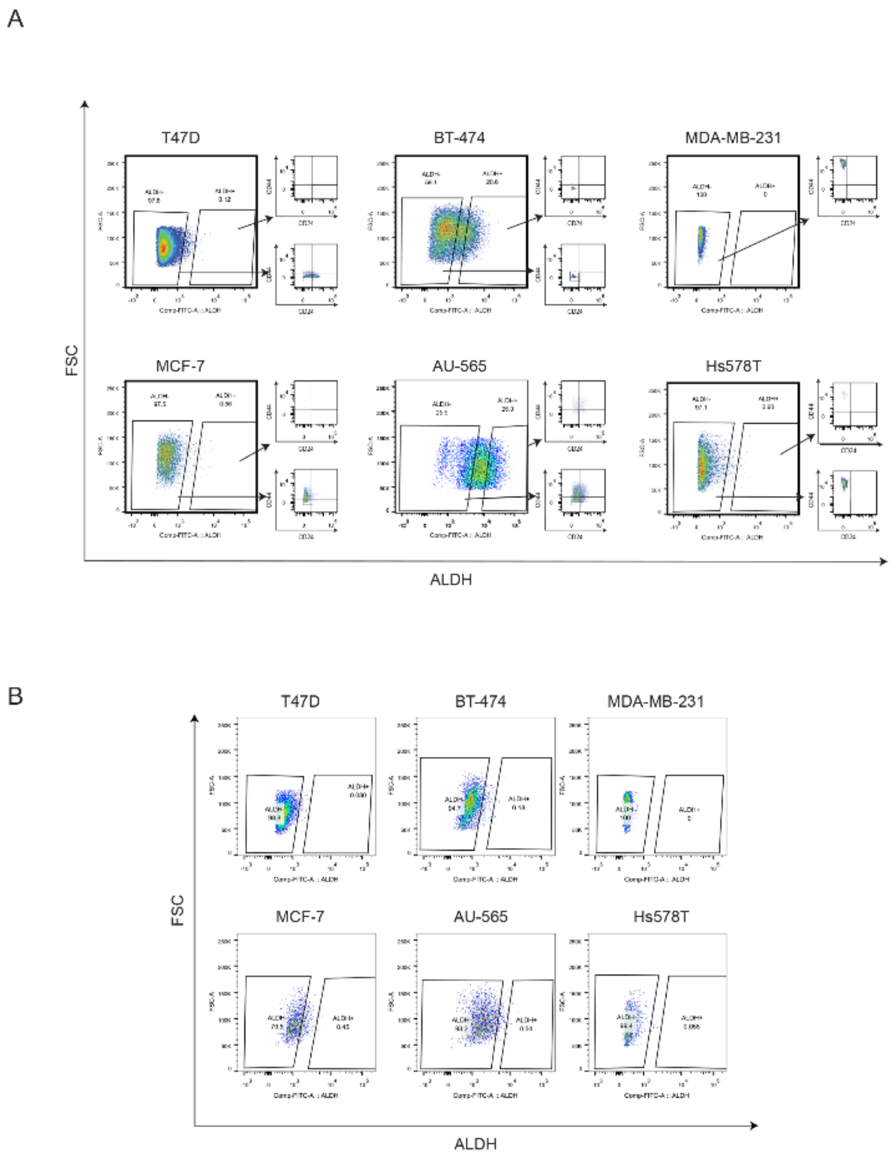

Figure S1: Gating strategy to isolate ALDH^{br}, CD44^{high}/CD24$^{-/low}$ and CD44$^{-/low}$/CD24$^{-/low}$ populations (A) ALDH^{br} cells were the highest in HER2-OE cell lines BT-474(~20%) and AU-565(~6%); CD44$^{-/low}$/CD24$^{-/low}$ in luminal T47D (~64%), MCF-7(57%), BT-474(~59%) and CD44^{high}/CD24$^{-/low}$ in triple negative cell lines Mda-MB-231 and Hs578T (~99%). Gating were set to DEAB inhibitor control(B).

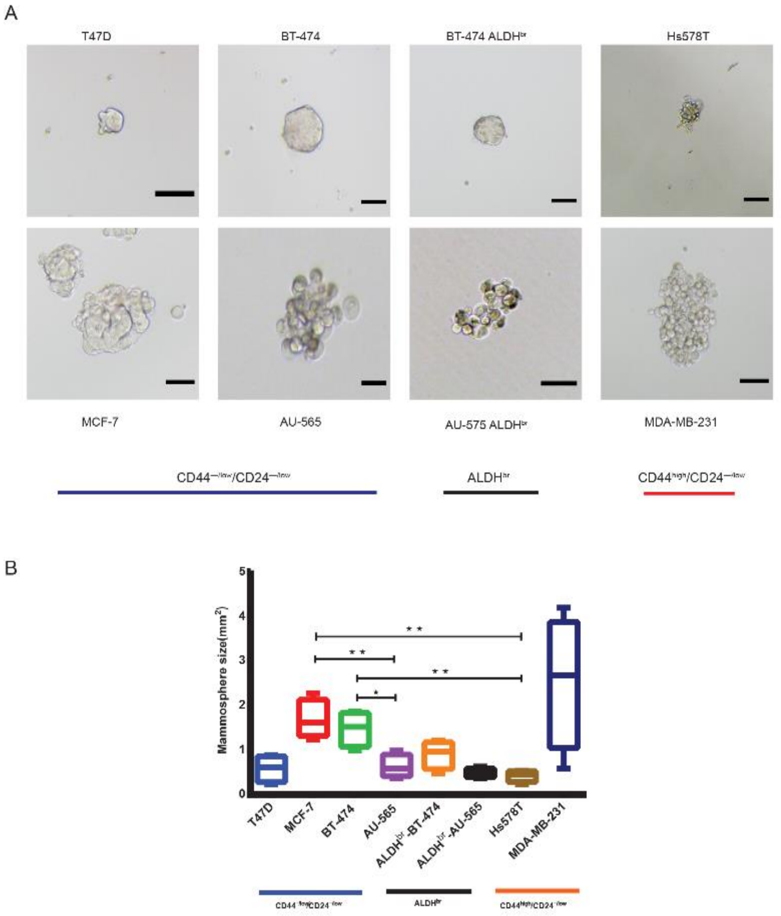

Figure S2: Luminal CD44$^{–/low}$/CD24$^{–/low}$ cells form tightly packed spheres

(A). Representative images of primary mammospheres grown in serum deficient DMEM medium after 7 days. All subpopulations formed discernable spheres by day 4 of culture. Luminal (T47D, MCF-7 and BT-474) spheres consist cells more tightly packed together. Triple negative (MDA-MB-231, Hs578T) and AU-565 cell lines formed aggregates of loosely packed cells. Scale bar = 100 µm. Sphere size comparison of CD44$^{–/low}$/CD24$^{–/low}$, CD44^{high}/CD24$^{–/low}$ and ALDH^{br} cell after 7 days in culture (B). CD44^{high}/CD24$^{–/low}$ cells from MDA-MB-231 formed the biggest aggregate spheres. CD44$^{–/low}$/CD24$^{–/low}$ cells from MCF-7 and BT-474 formed significantly larger spheres than CD44^{high}/CD24$^{–/low}$ and ALDH^{br} cells from AU-565 and hs578T cell lines.). The data represent the mean ±S.D. from three independent experiments performed in triplicate. *$P < 0.05$, **$P < 0.01$

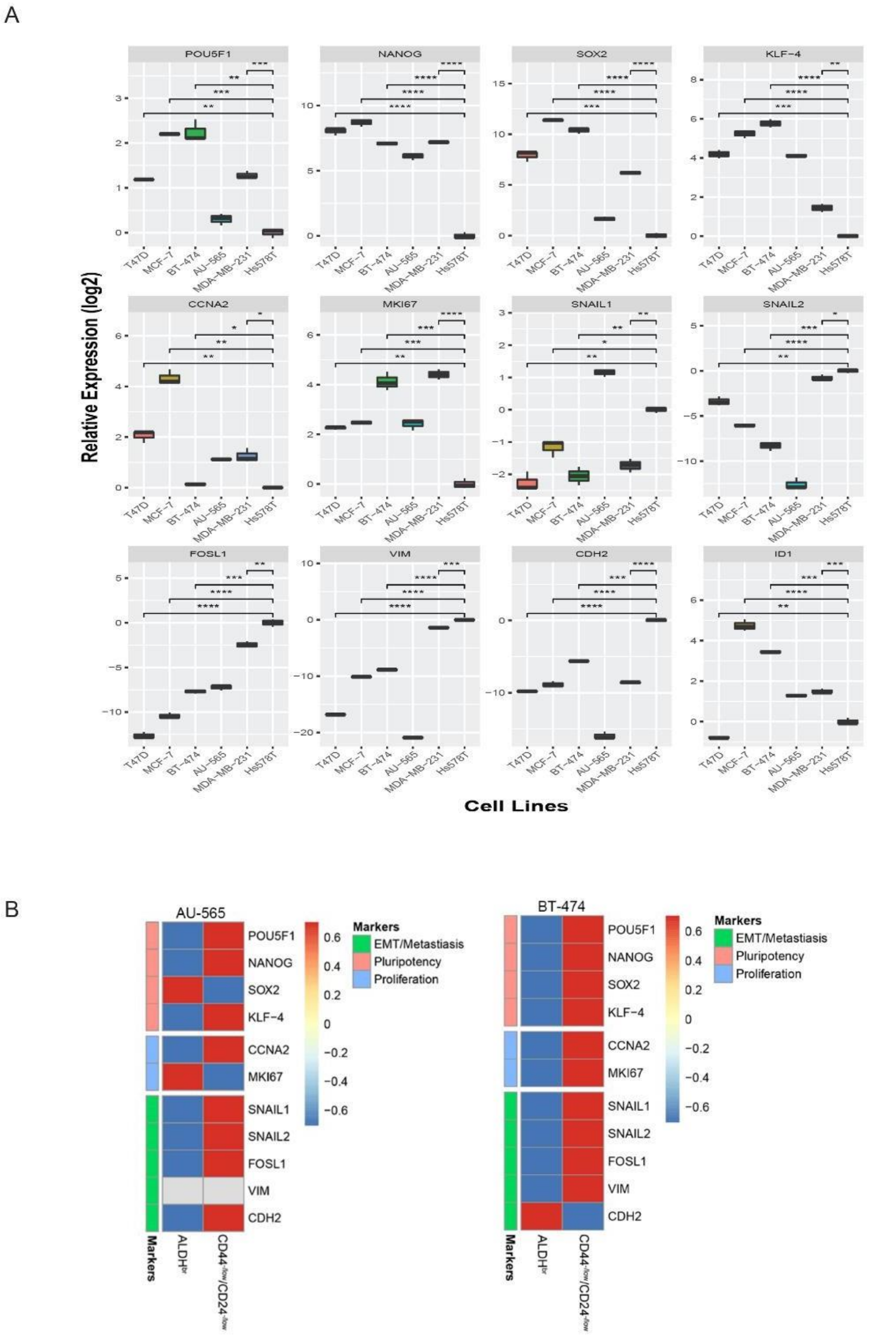

Figure S3: Relative expression of markers for pluripotency, proliferation and EMT/metastasis

(A) CD44$^{-/low}$/CD24$^{-/low}$ luminal cells show overexpression of metastasis and proliferation marker but low expression of EMT markers compared to CD44^{high}/CD24$^{-/low}$ cells from triple negative cell line Hs578T. Both the triple negative cell lines show comparable expression of EMT genes. Hs578T has significantly lower expression of NANOG and MKI-67. (B) CD44$^{-/low}$/CD24$^{-/low}$ cells from both HER2-OE cell lines BT-474 and AU-565 show marginal overexpression of metastasis, proliferation and EMT markers comparative to ALDH^{br} cells from their respective cell lines. All comparisons relative to bulk gene expression of respective cell lines. The data represents relative fold change (log_2) from three independent experiments. Fold change relative to Hs578T expression. *$P<0.05$, **$P<0.01$, ***$P<0.001$, ****$P<0.0001$
